# Supplementary material for: A Critical Gap in Seagrass Protection: Impact of Anthropogenic Off-Shore Nutrient Discharges on Deep Posidonia oceanica Meadows
Source: Plants (Basel). 2023 Jan 19;12(3):457. doi: 10.3390/plants12030457 (PMC9921266; doi:10.3390/plants12030457)
Supplement: Supplementary file 1 [file plants-12-00457-s001.zip › plants-1929439-supplementary.pdf]

Supplementary material

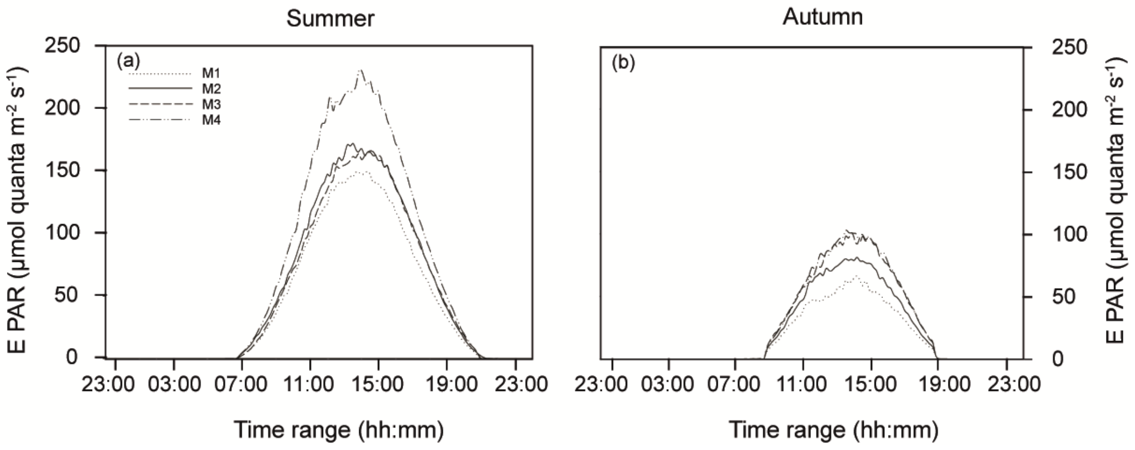

Figure S1. Daily PAR irradiance (E PAR; a and b) curves measured at the canopy level at the four sampling sites (M1–M4).

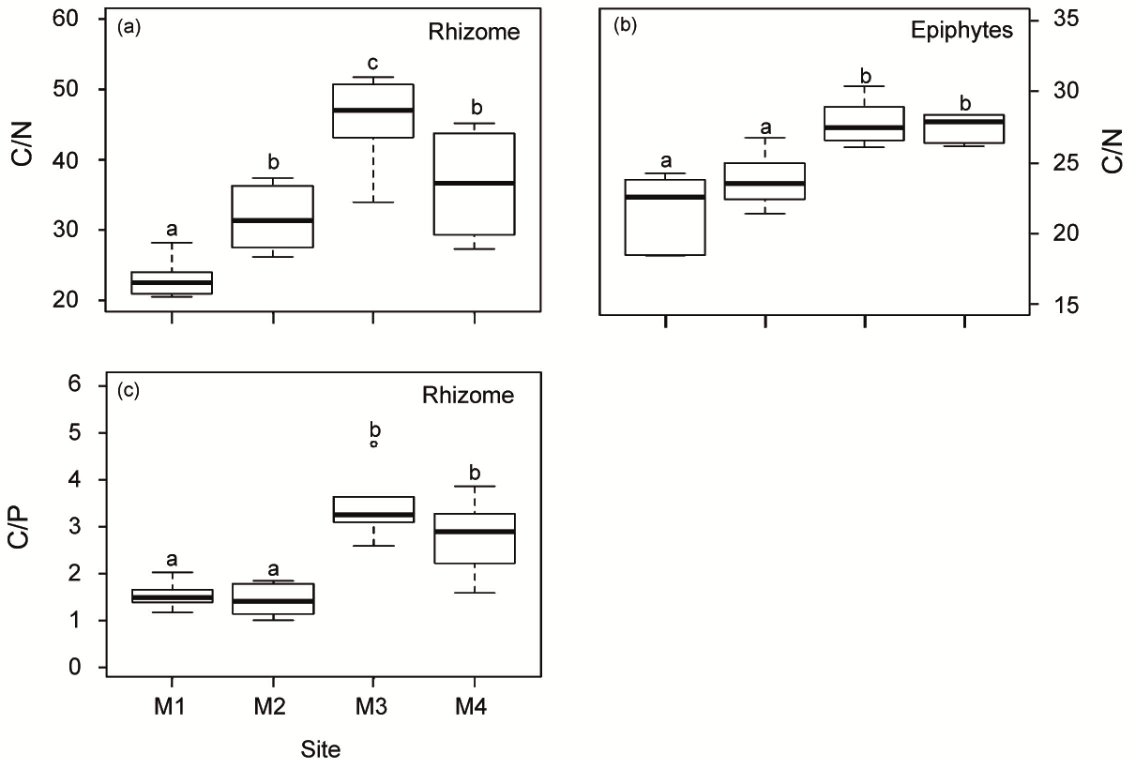

Figure S2. C/N ratio (a-b) and C/P ratio (c) measured in epiphytes and/or rhizome in autumn at the four sampling sites (M1–M4). Different letters denote significant differences among sites (Tukey test, p<0.05).

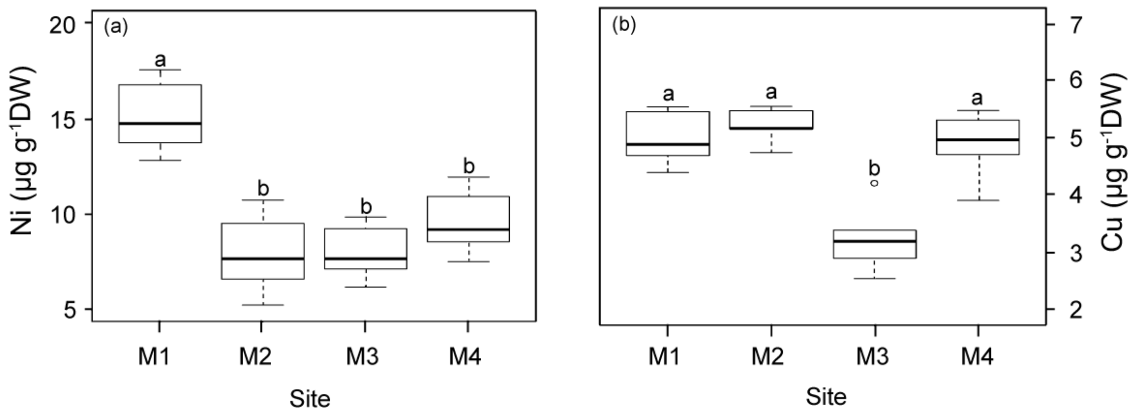

Figure S3. Metal content (Ni and Cu; a and b, respectively) measured in the rhizome in autumn at the four sampling sites (M1–M4). Letters denote significant differences among meadows (Tukey test, $p<0.05$).

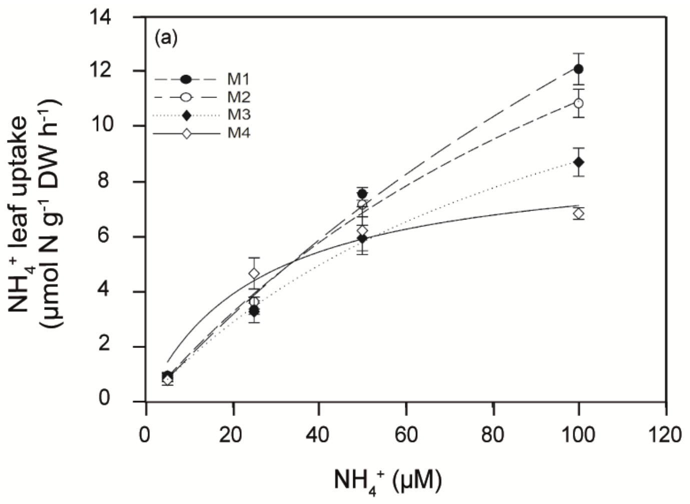

Figure S4. Leaf uptake kinetics for NH_4^+ at increasing concentration determined in *P. oceanica* in autumn at the four sampling sites (M1–M4). Uptake kinetics was adjusted by the Michaelis-Menten model. Values are means (n=6) and standard error.

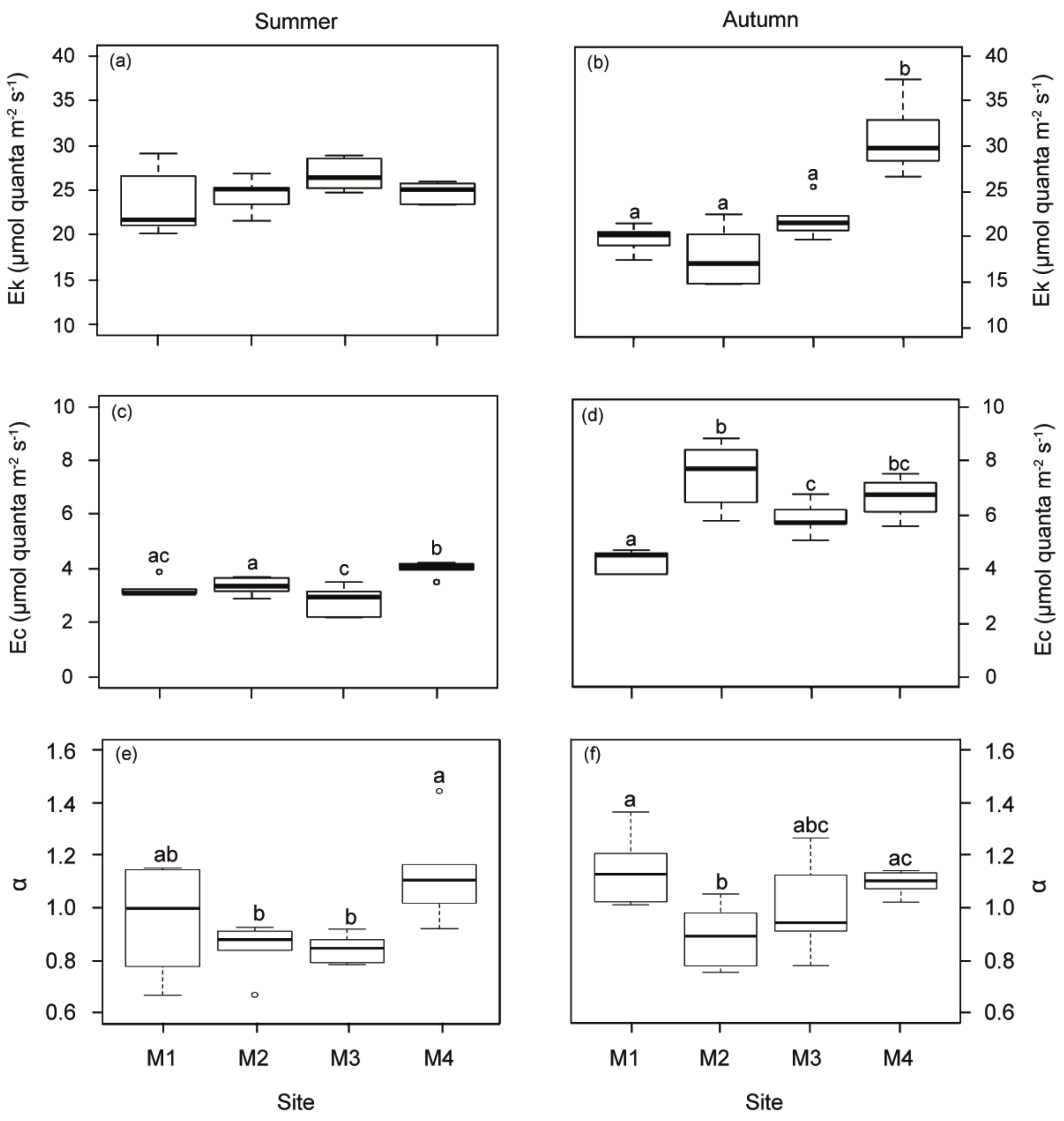

Figure S5. Saturation irradiance (Ek; a and b), compensation irradiance (Ec; c and d) and photosynthetic efficiency (α; e and f) in summer and autumn (left and right, respectively) at the four study sites (M1–M4). Different letters denote significant differences among sites (Tukey test, $p<0.05$).

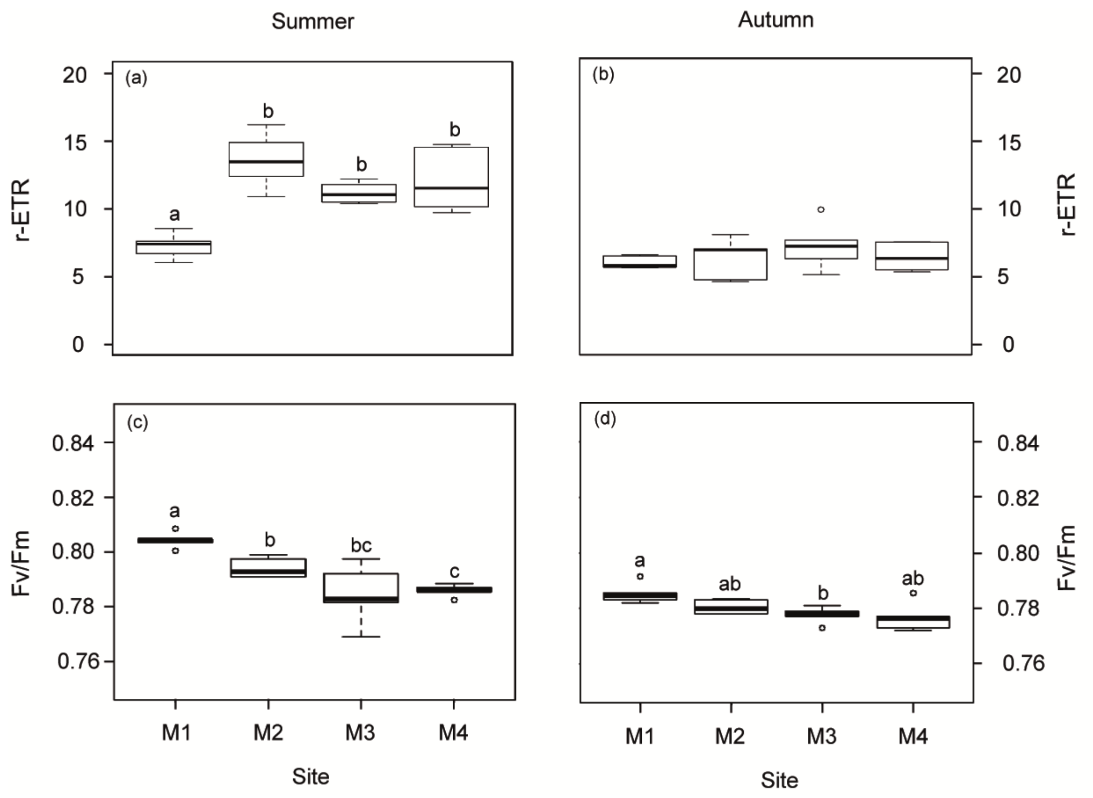

Figure S6. Chlorophyll fluorescence emission measured on *P. oceanica* leaves. Relative electron transport rate (r-ETR; a and b) and maximum quantum yield of photosystem II (Fv/Fm; c and d) in summer and autumn (left and right, respectively) at the four study sites (M1–M4). Different letters denote significant differences among sites (Tukey test, $p<0.05$).

Table S1. Percentage of variance explained by axes I and II, and eigenvalues obtained for biotic indicators of external nutrients enrichment, and abiotic factors included in the Principal components analysis (PCA).

**Variance**	**Axis I**	**Axis II**
	84.80 %	11.27 %
**Eigenvalue**	**Axis I**	**Axis II**
Ni	-0.5516	-0.5464
Cu	-0.2150	-0.9070
% P	-0.6171	-0.5445
% N rhizome	-0.7142	-0.6742
δ^{15}N rhizome	-0.9158	0.2661
C/N rhizome	0.7255	0.6827
N epiphytes	-0.9699	-0.1287
δ^{15}N epiphytes	-0.9491	-0.3112
C/N epiphytes	0.8960	0.4384
C/P rhizome	0.7708	0.6081
V_{max}	-0.9991	0.0425
K_m	-0.9874	0.1579
α_{uptake}	0.8205	-0.5708
r-ETR	0.4496	0.8932
Fv/Fm	-0.9341	-0.3197

OM %	-0.9236	-0.0961
DPPF	0.8438	0.2891
Distance	0.9567	-0.2477
